# Gradient 3D Printed PLA Scaffolds on Biomedical Titanium: Mechanical Evaluation and Biocompatibility

**DOI:** 10.3390/polym13050682

**Published:** 2021-02-24

**Authors:** Diana V. Portan, Christos Ntoulias, Georgios Mantzouranis, Athanassios P. Fortis, Despina D. Deligianni, Demosthenes Polyzos, Vassilis Kostopoulos

**Affiliations:** 1 Department of Mechanical and Aeronautics Engineering, Laboratory of Biomechanics and Biomedical Engineering, University of Patras, 265 04 Patras, Greece; portan@upatras.gr (D.V.P.); deliyian@upatras.gr (D.D.D.); 2 Department of Mechanical Engineering and Aeronautics, Applied Mechanics & Vibrations Laboratory, University of Patras, 265 04 Patras, Greece; christosntoulias@yahoo.gr (C.N.); gmantzou86@gmail.com (G.M.); polyzos@upatras.gr (D.P.); 3 Panarcadian General Hospital, B’ Orthopedics, 22100 Tripoli, Greece; apfortis@yahoo.com

**Keywords:** 3D printed scaffolds, scaffold mechanical evaluation, PLA scaffolds, scaffold biocompatibility

## Abstract

The goal of the present investigation was to find a solution to crucial engineering aspects related to the elaboration of multi-layered tissue-biomimicking composites. 3D printing technology was used to manufacture single-layered and gradient multi-layered 3D porous scaffolds made of poly-lactic acid (PLA). The scaffolds manufacturing process was optimized after adjusting key printing parameters. The scaffolds with 60 μm side length (square-shaped pores) showed increased stiffness values comparing to the other specimens. A silicone adhesive has been further used to join biomedical titanium plates, and the PLA scaffolds; in addition, titania nanotubes (TNTs were produced on the titanium for improved adhesion. The titanium-PLA scaffold single lap joints were evaluated in micro-tensile testing. The electrochemical processing of the titanium surface resulted in a 248% increase of the ultimate strength in the overlap area for dry specimens and 40% increase for specimens immersed in simulated body fluid. Finally, the biocompatibility of the produced scaffolds was evaluated with primary cell populations obtained after isolation from bone residual tissue. The manufactured scaffolds present promising features for applications in orthopedic implantology and are worth further.

## 1. Introduction

Conventional orthopedic implants are made of metals (titanium and titanium alloys). These confer high mechanical strength and fatigue resistance; however, the so-called ‘stress shielding’ effect is the main cause that determines manufacturers to aim at replacing them [1]. In order to create an appropriate environment for tissue ingrowth and a high biointegration rate of the implant, the joining of functional materials with properties similar to the natural tissue is the ultimate goal. Biomimetic scaffolds for bone tissue engineering, with multi-level lamellar structures to mimic natural bone tissue and play the role of scaffolds to treat bone tissue defects have been previously proposed [2]. Multi-layered materials for tissue reconstruction are of particular interest since they mimic well the natural tissue. The development of 3D scaffolds consisting of stacked multi-layered porous sheets featuring microchannels has been investigated for over a decade [3]. Porous materials are preferred because they allow the diffusion of nutrients and signaling products between the layers, whereas the micro channels facilitate nutrient supply on all layers as they provide space for the culture medium to be perfused throughout the scaffold. In orthopedic implantology, the key features of the materials are related to the mechanical properties and the osseointegration. It has been stated that coating the orthopedic metal is essential to develop novel biomaterial surfaces that promote better osseointegration, thus reducing implant failure and revision surgeries. Surface modification strategies using natural biopolymers on titanium have been recently proposed [4,5].

Despite the existence of several novel technologies, the use of conventional biomaterials (monophasic or composites) for interface tissue engineering has certain limitations in recreating the structural organization at the junction of different tissue types [6]. For instance, while numerous studies relate polymeric scaffolds to orthopedic applications [7], few solutions exist for the addition of the coating to an orthopedic metal. Often, biomaterials performance is evaluated from biological viewpoint, mainly biocompatibility. There are a lack of multidisciplinary studies including both mechanical and biological assessments in order to provide key engineering and medical solutions for existing issues. The application of polymeric scaffolds as coatings for the functionalization of metallic implants is an attainable, but still complicated solution. Thin electrospun coatings have been recently added on metals [8,9]; the method is dependent on the manufacturing process of the scaffolds and limited with respect to specimen size, shape, and production time. In other cases, adhesive mediated joining of the parts is the only solution. However, such a structure involves several interfaces/interphases and should be resist to complex mechanical loads in a physiologic environment. Further on, the overall structure should be non-toxic and highly biocompatible [10,11].

3D scaffolds mimic the properties of natural tissues and provide a template for cell development while stimulating tissue formation in vitro. Many requirements should be accomplished to obtain ideal scaffolds, such as biocompatibility and controlled biodegradability, to promote cellular interactions and tissue development and to possess appropriate mechanical and physical properties. One of the promising technologies used in scaffolds manufacturing is 3D printing, which allows to directly print porous materials with designed shape, controlled chemistry and interconnected porosity [12]. 3D printing, as an advanced fabrication technology, has received considerable attention due to its high precision, freedom in selecting customized geometry and personalization, as well as because it allows the processing of several thermoplastics for bone tissue engineering [13].

Several studies highlighted that PLA and PLA-based composites show superior mechanical behavior compared to other investigated thermoplastic polymers [14,15]. PLA has been previously used to manufacture originally designed and possibly suitable scaffold structures for bone tissue engineering, even with ordinary 3D printers [16]. Great advantages associated to PLA are that it is non-toxic, and is approved by the FDA for biomedical use. Besides that, its architectural features may be tailored according to the mechanical and biological requirements associated to specific applications. In addition, the mechanical behavior of the PLA scaffolds can be improved by adjusting pores dimension and distribution. Likewise, pore size is a key parameter to obtain substrates that stimulate tissue ingrowth, and 3D printed technologies allow for the design of gradient properties along scaffold thicknesses. Gradient materials that biomimic the natural interfaces between tissues are of high biomedical interest nowadays. Engineering interface tissues is a complex process, which requires a combination of specialized biomaterials with spatially organized composition.

Within the present research, 3D printed PLA scaffolds were added on biomedical titanium plates. Optimum 3D printed PLA single-layered and gradient multi-layered scaffolds with square-shaped pores with side lengths between 60 to 100 μm were manufactured after adjusting the key printing parameters. The stiffness of the produced scaffolds was evaluated. In addition, single lap dissimilar joints were manufactured from PLA scaffolds and titanium sheets, by using a silicone-based adhesive. The interphasial adhesion between the dissimilar parts has been evaluated in micro-tensile testing before and after immersion in a simulated body fluid (SBF). The interphase between the two materials has been strengthened with titanium dioxide nanotubes (TNTs) synthesized on the titanium surface. Finally, the biocompatibility of the produced scaffolds has been evaluated with primary bone cells differentiated into osteoblasts.

## 2. Materials and Methods

### 2.1. Materials

Natural-PLA with a diameter of 1.75 mm from Innofil3D (Emmen, The Netherlands) has been used for the manufacturing of the compact and porous PLA specimens. 99.9% purity titanium sheets with 0.5 mm thickness provided by Sigma Aldrich, Hamburg, Germany were used as adherends in the dissimilar joints. A commercial silicone-based adhesive (PolyMax Crystal, Bison, Rotterdam, The Netherlands) has been used to join the PLA scaffolds and the titanium foil.

### 2.2. Manufacturing Methods

#### 2.2.1. Adjustment of Key Printing Parameters

A 3DISON AEP desktop 3D printer (ROKIT, Seoul, Korea) based on Fused Filament Fabrication (FFF) was used to print the PLA. The software was programmed to allow the fabrication of scaffolds with square shaped pores having side lengths between 60 to 100 μm, in single or multi-layered structures. In the case of multi-layered scaffolds, the side lengths of the pores were increased from the basic layer towards the upper level of the specimen. Two- or four-layer structures (60–70, 60–80 and 60–70–80–90 μm) were printed. The key parameters: nozzle temperature (*T*_nozzle_, °C), chamber temperature (*T*_chamber_, °C), printing speed (V, mm/s), printing resolution along ‘z’ axis (R, mm), bed temperature (*T*_bed_, °C), raster angle (*R*_infill_, deg), density (*D*, %), were adjusted to allow the manufacturing PLA specimens with optimum properties. The selection of an ideal combination of parameters was made according to the output from the tensile testing of compact PLA specimens.

#### 2.2.2. Titanium Functionalization

The electrochemical anodization method was employed for the surface functionalization of the titanium foil. The purpose was to confer a nanoarchitecture to the surface and assure an increased contact area with the silicone-based adhesive, for an improved adhesion. The selected anodization procedure to process the titanium is simple and cost-effective and it has been previously described [17,18]. The electrochemical anodization led to the formation of a highly organized and very thin layer of titania nanotubes (~500 nm height), parallel in between them and perpendicular to the titanium surface, with diameters between 80 and 100 nm each.

#### 2.2.3. Single Lap Joints

**Figure 1:**
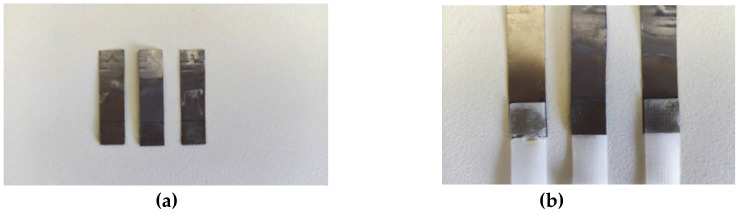
(**a**) Anodized titanium adherends and (**b**) Single lap joints made of poly-lactic acid (PLA) scaffolds and anodized titanium adherends.

Dissimilar single lap joints made of PLA scaffolds and titanium were manufactured using a commercial silicone-based adhesive. Two sample categories were tested: (1) joints made of titanium and PLA scaffold adherend and (2) joints with electrochemically modified titanium and PLA scaffold adherend (Figure 1). The dimensions of the titanium and the PLA adherends were: 4 cm length, 1 cm width, 0.5 mm thickness, 1 cm overlap length, and 1 mm overlap thickness. The pore side length of the PLA scaffolds was 100 μm. The adherends were joined with the adhesive by applying a constant pressure of 300 Pa on the overlap region for 8 h.

### 2.3. Characterization

#### 2.3.1. Optical Images and Pore Volume

A Jeol, JSM-6300 scanning microscope (SEM, Jeol, Tokyo, Japan) was used to obtain micrographs of the PLA scaffolds. Pore volume was calculated according to the data obtained from the slicing software of the 3D printer. For instance, in the case of a scaffold with pore side length of 60 μm, the data and calculations were as following:

(1) Filament diameter: 1.75 mm
(2) Filament used to 3D print the scaffold (h): 23.75 mm
(3) The volume of the printed scaffold and the theoretical volume are:
  V_printed scaffold_: π × r^2^ × h = 57.1 mm^3^, where ***π*** is 3.14; *r* is the radius and *h* the height
  V_theoretical_: 85 mm^3^ (is a solid scaffold 10 × 10 × 0.85)
(4) The calculated porosity is thereof calculated:
  V_theoretical_ − V_printed scaffold_ = V_porosity_ = 27.9 mm^3^
  V_porosity_ (%) = 32.82%

#### 2.3.2. Mechanical Evaluation

**Figure 2:**
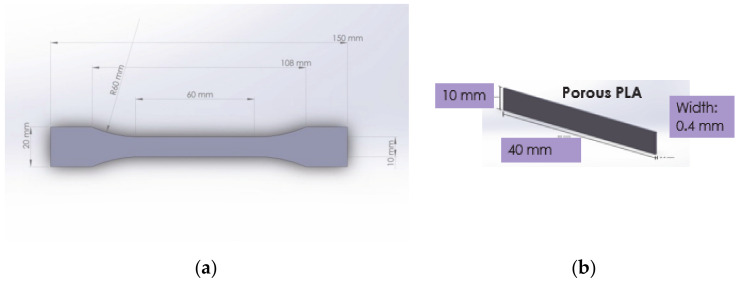
Dimensions of: (**a**) Compact PLA specimens prepared according to ISO 527-2 and (**b**) Porous PLA specimens for micro-tensile testing with a Metravib DMA 50machinE

Compact PLA specimens were tested in tensile mode with the purpose to adjust the printing parameters. Specimen dimensions respected ISO 527-2 (Figure 2a). The testing machine was an INSTRON-8872 (Instron, Norwood, MA, USA). The outcome of the tensile testing is presented Section 3. Further on, the stiffness of single and multi-layered porous PLA specimens was measured with a Metravib DMA 50 machine (Metravib Desisgn, Limonest, Rhone, France), in static tensile, at 37 °C. The specimen dimension were as indicated in Figure 2b.

**Figure 3:**
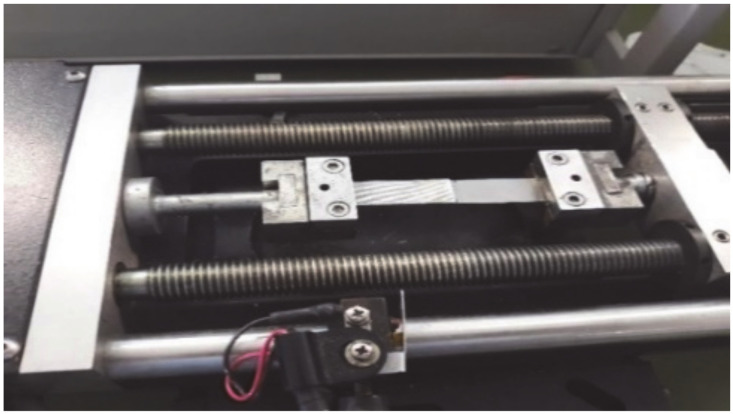
Setup of MiniMat 2000 Machine for the tensile testing of titanium-PLA joints.

Micro-tensile tests were performed on dissimilar PLA-titanium single lap joints (Figure 3) using a MiniMat 2000 Machine from Rheometric Scientific (Piscataway, NJ, USA). For each sample category, three specimens were tested.

The four sample categories were: (1) Single lap dissimilar joints with titanium and PLA scaffolds; (2) Single lap dissimilar joints with electrochemically modified titanium and PLA scaffolds; and the same two after immersion of the joints in a simulated body fluid (SBF) for 8 h. The simulated body fluid consisted in a Ringer’s solution made of 8.5 g/L NaCl, 0.25 g/L KCl, 0.22g/L CaCl_2_, 0.15 g/L NaHCO_3_, with a pH value of 7.8 ± 0.1.

#### 2.3.3. Evaluation of Substrates Biocompatibility

The dimension of the tested samples was 1 × 1 cm^2^. The following substrates were used: (1) Tissue culture polystyrene (TCP) as a reference material; (2) Single layer PLA scaffold with 60 μm pore side length; (3) Single layer PLA scaffold with 100 μm pore side length; (4) Four-layer PLA scaffold with 60–70–80–90 μm pore side length; (5) Titanium, and (6) TNTs. The biocompatibility of the manufactured substrates was compared to previously reported electrospun materials [19]. The substrates were cleaned with ethanol and sterilized under UV light.

**Figure 4:**
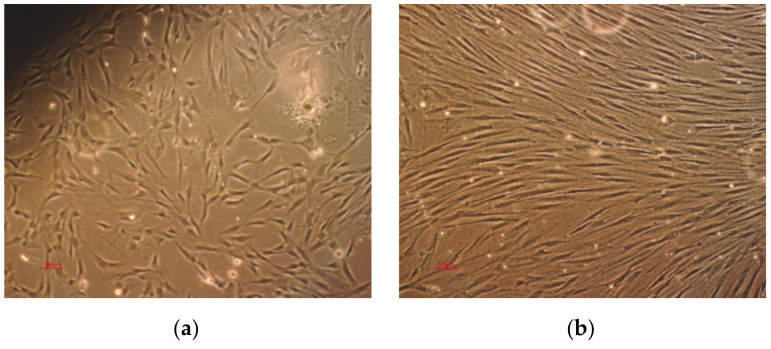
Differentiated osteoblasts: (**a**) second passage and (**b**) at confluency.

Human bone marrow cells were isolated with the patient consent from residual bone obtained from a 50 year old male undergoing total hip arthroplasty surgery. The cells were differentiated into osteoblasts using an appropriate cell culture medium, as described in [20]. The cell suspension was cultured until confluence. During multiplication, proliferation and while seeded on TCP substrate, osteoblasts (Figure 4) were observed with an inverted optical microscope (Nikon Diaphot, Tokyo, Japan).

A cell population containing ~6 × 10^4^ osteoblasts were seeded on each testing substrate. This number of cells has been considered the most appropriate for the type of materials involved in the present research and was selected according to the output of a previous study [21].

The Alkaline Phopsphatase (ALP) and the Total Protein (TP) levels were measured in osteoblasts after 1 and 3 days of incubation on the substrates. ALP enzyme activity was assessed with the P0757L kit from BioLabs. Bradford Protein Quantification kit (Bertin Pharma, Montigny le Bretonneux, France) was used to quantify the total protein level. An Infinite F200PRO UV/visible Spectrometer was used to detect the ALP and the TP levels at 405 nm, respectively 600 nm wavelengths. The experimental data were analyzed using SPSS 14.0 software (SPSS, Armonk, New York, NY, USA). The level of significance was calculated using Student’s test for single comparisons and ANOVA for multiple comparisons. Statistical significance was assumed at a 95% confidence limit or greater (*p* < 0.05). For SEM imaging, cells were deposited on substrates for 12 h and further on fixed with formaldehyde, for 15 min.

## 3. Results

### 3.1. Microscopy and Pore Volume

**Figure 5:**
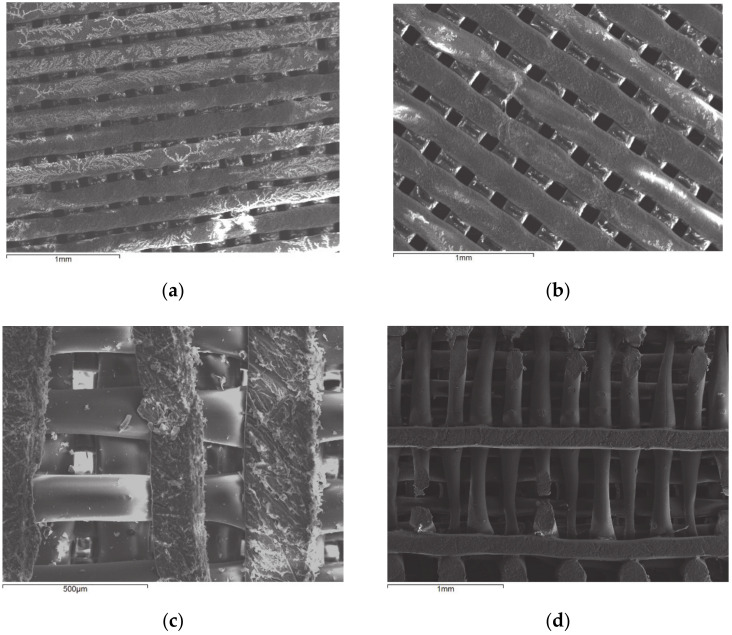
Micrographs of scaffolds with pore side length:(**a**) 60 μm, (**b**) 100 μm, (**c**) 100 μm, and (**d**) 100 μm after immersion in simulated body fluid (SBF) for 8 h.

Comparing Figure 5a,b, one may observe that the scaffold with 60 μm pore side length is denser than the scaffold with 100 μm pore side length. Both architectures are highly organized. It has been previously demonstrated how architectural design of 3D printed scaffolds can improve in vivo outcomes. More exactly, it has been shown that manipulating pore size and permeability in 3D printed scaffold architecture provides a useful strategy for enhancing bone regeneration outcomes [22]. Figure 5c depicts the structure of the PLA fiber at high magnitude. In Figure 5d, the breakage of the fiber as a result of immersion in SBF for 8 h can be observed.

The theoretical pore volume was calculated for the single and multi-layered PLA materials. The porosity volume can be observed in Table 1.

The lowest pore volume was found, as expected, in the case of the single-layered scaffold with 60 μm side length of the square shaped pores. The addition of another layer to the scaffold, with longer side length (70 μm) resulted in a ~2% increase of porosity volume. Thus, the double-layered scaffold with 60–70 μm side lengths of the pores has ~34% porosity and the scaffold with 60–80 μm side lengths of the pores has ~36% porosity compared to the single-layer scaffold with 60 μm side length, that has only 32% porosity. The highest value of the pore volume can be observed in the case of the single-layer scaffold with 100 μm side length. This scaffold can be considered the most appropriate for applications that require fluid penetration, rather than to support the ingrowth of cell networks.

### 3.2. Mechanical Evaluation: Adjustment of Printing Parameters

The adjustment of the printing parameters is very important to obtain scaffolds with optimum mechanical properties. Some factors that can change the strength of 3D printed pieces are the infill percentage, the layer height, the print orientation, the extruding temperature, and the building speed [23]. In the present investigation, several printing parameters were modified to achieve an optimum combination and thus improve the manufacturing process (Table 2). The samples were manufactured from compact PLA. As observed in Table 2, between the tested specimens, specimen number 2 had highest Young modulus and tensile strength; the used parameters were as following: 190 °C nozzle temperature, 40 °C chamber temperature, 30 °C bed temperature and low printing speed (20 mm/s).

This may be explained assuming that the high jump in temperatures between the nozzle (190 °C), the chamber, and the bed (40, 30 °C) leads to a fast cooling of the thermoplastic; this process maintains the material in an amorphous state since the fast cooling does not permit its crystallization. This in turn, determines an improvement in the mechanical properties of the printed samples. The printing parameters used to manufacture Specimen 2 were applied to elaborate single and multi-layered PLA scaffolds with different pore side lengths (60, 70, 80, 90, 100 μm).

### 3.3. Micro-Tensile Testing of Scaffolds

**Figure 6:**
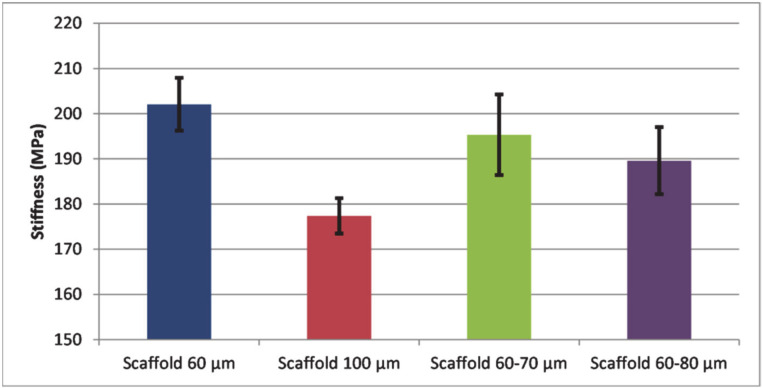
Stiffness of scaffolds with different pore side lengths: single layer (60 μm, blue), single layer (100 μm, orange), double layer (60 and 70 μm, grey) and double layer (60 and 80 μm, yellow).

Since both strength and stress-strain distributions throughout the scaffold depend on its internal architecture, it is important to understand how changes in architecture influence these parameters. Ideal pore dimension to assure both mechanical strength and human cell development should be achieved. Due to an increased mass of the structure, scaffolds with smaller pores are generally more robust. In a recent study by Velioglu et al. [24] it was shown that compressive moduli of scaffolds decreased with increasing pore dimensions. Further on, it was found that an ordered structure confers higher values of the mechanical properties to the 3D printed porous materials, minimizing negative effects of a high porosity on strength. Generally, small pore size was beneficial for the mechanical strength of porous scaffolds [25]. As observed in Figure 6, the lowest stiffness value was found in the case of a single layer scaffold with 100 μm pore diameters.

This confirms the results found in the above-mentioned studies. Further on, higher stiffness values correspond to higher scaffold masses. Therefore, the single-layered scaffold with 60 μm pore side length presents the highest stiffness, followed by the double-layered scaffolds with 60 and 70 μm pore side lengths and double-layered scaffolds with 60 and 80 μm pore side lengths.

### 3.4. Micro-Tensile Testing of Titanium-PLA Single Lap Joints

**Figure 7:**
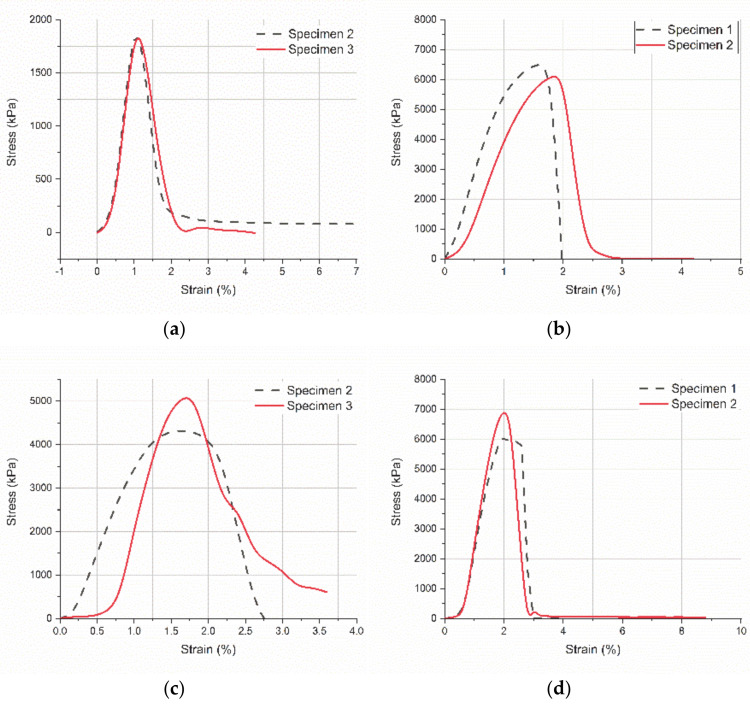
Stress-strain curves for single lap joints (micro-tensile tests): (**a**) Titanium-PLA, (**b**) TNTs-PLA; (**c**) Titanium-PLA after 8 h immersion in SBF, and (**d**) TNTs-PLA after 8 h immersion in SBF.

The overlap area in single lap joints is a key region where the progress of several mechanical phenomena may be observed in detail. Joining two dissimilar materials in composite systems to evaluate their mechanical compatibility by analyzing mechanical parameters at the interface is extremely useful to improve structures performance in specific applications. In the present study, pure titanium and PLA scaffolds were joined using a commercial silicone adhesive. To improve the adhesion strength, titanium was electrochemically processed, and a thin nanometer scale TNTs layer was manufactured on its surface. The increased surface area of the titanium adherend in contact with the adhesive considerably improved the strength of the joint. As observed in Figure 7, the stress-strain curves indicated a different breakage mode and considerable increased values of the tensile strength when TNTs are present at the interface.

**Figure 8:**
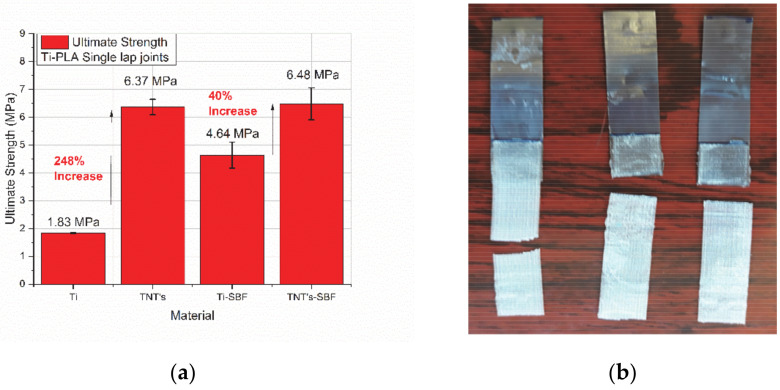
(**a**) Ultimate strength after micro-tensile testing: Ti: titanium-PLA joints, TNTs: titanium dioxide nanotubes -PLA joints, Ti-SBF: titanium-PLA joints after 8 h SBF, TNTs-SBF: titanium dioxide nanotubes -PLA joints after 8 h immersion in SBF and (**b**) PLA yielding mode in joints with TNTs on the titanium adherend.

For dry specimens, the tensile strength of titanium-PLA joints (Figure 7a) is more than three times lower–2000 kPa than the tensile strength of TNTs-PLA joints-6500 kPa (Figure 7b). Further on, when comparing the profile of the curves for the joints with TNTs (Figure 7a,c) vs. those without TNTs (Figure 7b,d), it may be seen that the necessary elastic strain energy to break the specimens during mechanical loading is much higher when TNTs are present on the titanium surface. Additionally, the titanium-PLA joint collapsed because the two adherends slept apart; in this case, the adhesive yielded. On the opposite, TNTs-PLA joint collapsed outside the overlap region, when the PLA specimen broke up (Figure 8b). All these prove the important contribution of the nanoscale architecture to the adhesion strength between the two adherends.

An interesting phenomenon can be observed when comparing dry titanium-PLA joints with wet titanium-PLA joints. After 8 h immersion in SBF, the joints present considerable increased tensile strength-5000 kPa (Figure 7c) comparing to the dry specimens-1900 kPa (Figure 7a). The mechanism is related to previously reported anomalous effects of the water absorption mechanism. Extensive work focused on water absorption mechanisms in epoxy resins has been made; this showed that the polymer network initially becomes denser with water uptake, resulting in an improved mechanical behavior. Further on, as time passes, the plasticizing effect becomes stronger, leading to a subsequent degradation of the mechanical properties [26]. The threshold to be reached differs depending on the polymer type and the liquid chemistry or temperature. This mechanism is not observed in the case of joints with TNTs at the interface (Figure 7b vs. Figure 7d). The reason is that toughness increases due to TNTs nanoarchitecture. Moreover, it has been reported that 100 nm diameter TNTs are hydrophobic [27,28] and thereof they block the water uptake in the overlap region.

Finally, when analyzing the diagram in Figure 8a, it can be observed that an increase of 318% of the ultimate strength of the joint takes place with the addition of TNTs at the interphase, in the case of dry specimens. Further on, the ultimate strength of the specimens after 8 h immersion in SBF is 40% higher in joints with TNTs at the interphase.

### 3.5. Biocompatibility

**Figure 9:**
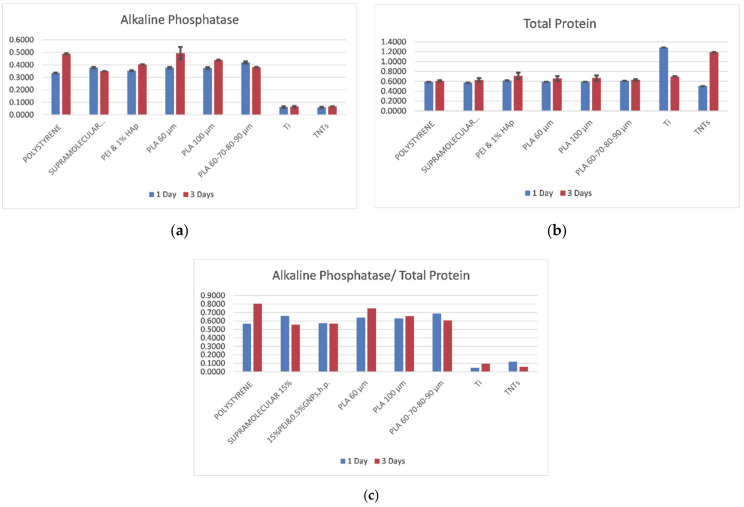
Marker levels expressed in cells seeded on different substrates, after 1 and 3 days of incubation: (**a**) Alkaline Phosphatase (**b**) Total Protein and (**c**) Alkaline Phosphatase/ Total Protein.

Basic biomarkers that show osteoblasts feedback to a substrate are the Alkaline Phosphatase (ALP) and the Total Protein (TP). Their levels are crucial within the first hours after cells are seeded on the material. ALP is an enzyme found in bone, with a role in hard tissue formation. It is the most widely recognized biochemical marker for osteoblast activity. The ALP in vitro indicates the degree of osteoblasts maturation, differentiation, and in vitro mineralization [29]. TP is also a known marker of the osteoblast phenotype and plays a key role in the quality of adhesion of the osteoblast on substrates [30]. Both ALP and TP parameters are considered crucial in the development of new biomaterials aimed to induce rapid and efficient osseointegration. ALP is related to osteoblasts maturation and differentiation, while TP is related to the degree of adhesion of the cells on the substrate. If their ratio-ALP/TP is high enough, it indicates a good proliferation induced by an appropriate adhesion, due to good cooperation between the cells and the underlying material [31]. In the diagrams in Figure 9, the ALP and TP levels expressed in cells after one and three days of incubation on the following substrates: tissue culture polystyrene (control material), PEI and GNPs (graphene nanoplatelets reinforced polyetherimide), PEI and HAp (hydroxyapatite reinforced polyetherimide), PLA 60 (polylactic acid with 60 μm pore side length), PLA 100 (polylactic acid with 100 μm pore side length), PLA 60–70–80–90 (multi-layered polylactic acid with 60–70–80–90 μm pore side length), Ti (pure titanium), and TNTs (titania nanotubes), may be seen.

It can be observed (Figure 9a) that the highest ALP level is expressed in cells on PLA, even compared to the reference material (TCP); this indicates that the 3D printed PLA is appropriate for osteoblasts differentiation. On the other hand, the difference between cells’ response to polymeric materials and to metallic substrates is extremely pronounced. It has been previously demonstrated that due to electrostatic mechanisms, titanium entraps cells and limits their development [32]. As clearly seen, all polymeric materials induce positive feedback of cells with 30 to 50% improvement in expressed ALP comparing to the metallic substrates (Figure 9a). Between all substrates, the PLA scaffold with 60 μm pore side length promotes ALP release by cells, especially after 3 days of incubation. However, longer incubation periods might show similar results on all side lengths of the pores, since it was reported that osteoblast lines indicated similar activity on several pore diameters [33].

In Figure 9b it can be seen that the protein level expressed in cells on polymeric materials is relatively constant for both one and three days of incubation. Extremely high total protein levels are released by cells on titanium after one day of incubation and on TNTs after three days of incubation. This is explainable and has been previously reported [20,32]. The cause is that when seeded on titanium substrate, cells develop in some hours to days and interconnect, which allow them to proliferate. On the other hand, when on TNTs, they anchor strongly and end up in necrosis.

No significant difference is detected in ALP and TP levels when cells are seeded on 60 μm pore side length PLA comparing to gradient PLA (60–70–80–90 μm pore dimeters). Finally, the diagram in Figure 9c gives a more complete icon on cells response when in contact with the investigated substrates. The higher the ALP/TP, the better since this indicates that cell adhesion to the substrate does not limit their development and differentiation. In vivo, the key function of osteoblasts is to interconnect and form tissue. In vitro, they adhere to the underneath platform and promote cytoskeleton expansion, which in turn enables proliferation. We clearly deduce by analyzing the diagram in Figure 9c that metallic substrates do not promote osteoblasts proliferation. Values of ALP/TP are almost undistinguishable after 3 days of cells incubation, when compared to the polymeric materials. Polyetherimide composites showed similar results independent of the type of the reinforcement (GNPs or HAp), and the values were comparable to those expressed in the case of the reference material (tissue culture polystyrene). Highest value of ALP/TP was expressed in cells that were seeded on 60 μm pore side length PLA, after three days of incubation. It has been recently found that cells cultured on small size granules exhibited lower cell viability, and higher osteopromotive ability, while a smaller granule size might be advantageous due to its greater bone regeneration potential [34]. Small dimensions of the substrate confer a higher contact area for cells to anchor and develop in the first hours after deposition. For short term incubation, the small pore diameter gives support for a robust interconnectivity of cells. For longer incubation time, as stated by others, the restriction in space decrease cells viability as they limit proliferation; thus, increased diameters promote cells proliferation for longer incubation periods [35]. Finally, increased values were detected for all PLA substrates after three days of incubation compared to the first day, showing that cells adapt fast to this substrate.

## 4. Conclusions

The general purpose of the present investigation was to elaborate strategies that enable the development of coatings on orthopedic implants. Several aspects of the overall performance of single and multi-layered porous PLA scaffolds were investigated. The 3D printing technique was used to manufacture single and multi-layered PLA scaffolds with square shaped pores having side lengths between 60 to 100 μm. The key manufacturing parameters have been adjusted to allow the elaboration of scaffolds with optimum mechanical properties. Several printing parameters were interplayed to find the ideal combination which leads to the formation of PLA scaffolds with optimum mechanical properties.

The concluding highlights of the present investigations are:

1. The most influent parameters in the 3D printing manufacturing process were the temperature of the printing components (nozzle, chamber, bed), and the printing speed, which should be low.
2. Single and double-layered scaffolds (60, 100, 60–70, and 60–80 μm side lengths) were tested in micro-tensile mode. The highest stiffness values were detected in single-layered samples with smaller pores (60 μm side length) due to an increased mass of their architecture. The study revealed that in terms of mechanical performance, single-layered scaffolds with small pore dimeter/side length present a more stable architecture for orthopedic implantology applications, where materials are subjected to complex loadings.
3. Scaffolds with 60–70 μm side lengths showed overall balance, with a moderate porosity and an improved stiffness (34% porosity and 190 MPa stiffness).
4. Small pore dimensions are preferred to manufacture synthetic orthopedic components. On the other hand, higher pore volume allows liquid diffusibility which is demanded in applications such as drug delivery systems. Scaffolds with larger pores or with gradient porosity along their thickness remain of interest when multi-functionality is targeted.
5. 3D printed PLA scaffolds with 100 μm side length of the pores were added on pure titanium and on TNTs (titania nanotubes) by using a silicone-based adhesive. Titanium-PLA single lap joints were tested in micro-tensile mode to observe the general performance and to measure the adhesion strength of the overlap area, before and after immersion in a simulated body fluid. A 248% increase in the ultimate strength of the dry joints was found with the addition of TNTs on titanium surface and a 40% increase of the ultimate strength was found for the same specimens after 8 h immersion in SBF.
6. The study showed that the adhesion of the coating/ scaffold to the medical titanium depends very much on the surface treatment of the metal and that highly organized, nano-level structures result in improved adhesion strength between the metal and the plastic coating.
7. A preliminary biocompatibility study indicated that the 3D printed PLA scaffolds induced positive behavior in primary osteoblasts cells comparing to all the other tested substrates (tissue culture polystyrene, metals, electrospun membranes). It was found that primary osteoblasts prefer smaller pore diameter/ side lengths in a single-layered structure, for short incubation periods. A 30% to 50% increase of expressed ALP was found in cells seeded on plastic substrates comparing to the metallic ones. This proves that the addition of plastic coatings on metallic implants is highly recommended.
8. In between all substrates, PLA with 60 μm pore side length promotes ALP release by cells, especially after 3 days of incubation. The superiority of this structure may be explained through the existence of an extended surface area for osteoblasts to adhere; at the same time, the small pore architecture allows cells to migrate and create tissue-networks along scaffold’ thickness. On the other hand, small dimeters may limit cell proliferation for long incubation periods.
9. Conventional titanium generally entraps cells and limits their development, which has been confirmed through the present investigation. The optimum solution is, therefore, the addition of a plastic scaffold on the metallic prosthesis.

## Figures and Tables

**Table 1:** Calculation of porosity volume for the various single and multi-layered PLA scaffolds.

Pore Side Length (μm)	Dimensions	Filament Length (mm)	*V*_scaffold_	*V*_PLA_ (%)	*V*_porosity_ (%)
**60**	10 × 10 × 0.85	23.75	85	67.22	**32.78**
**60–70**	10 × 10 × 0.45	12.16	45	65.02	**34.98**
**60–80**	10 × 10 × 0.45	11.87	45	63.48	**36.52**
**60–70–80–90**	10 × 10 × 0.85	23.46	85	66.40	**33.6**
**100**	10 × 10 × 0.85	21.5	85	60.84	**39.16**

**Table 2:** Key printing parameters and their effect on the E and the σ of compact PLA specimens.

Sample No.	*T*_nozzle_(°C)	*T*_chamber_(°C)	*V*(mm/s)	*R*(mm)	*T*_bed_(°C)	*R*_infill_	*D*(%)	*E* (GPa)	Tensile strength (MPa)
**0**	220	40	60	0.12	80	±45	100	2.45	33.88
**1**	220	40	20	0.1	80	0/90	90	2.49	34.30
**2**	**190**	**40**	**20**	**0.12**	**30**	**0/90**	**100**	**2.78**	**38.82**
**3**	190	40	60	0.1	30	±45	90	2.06	23.10
**4**	220	30	20	0.1	30	±45	100	2.72	36.99
**5**	220	30	60	0.12	30	0/90	90	2.42	33.33
**6**	190	30	60	0.12	80	0/90	100	2.63	36.53
**7**	190	30	20	0.1	80	±45	90	2.31	31.37

## Data Availability

All data are available and can be obtained upon request, from the authors.
